# Socioeconomic variation in the relationship between cardiac rehabilitation participation and clinical outcomes: a systematic review

**DOI:** 10.1136/openhrt-2025-003698

**Published:** 2025-12-07

**Authors:** Deborah Manandi, Julie Redfern, Qiang Tu, Abigail Ying Jie Chang, Nashid Sabrina Hafiz, Dion Candelaria, Karice Hyun

**Affiliations:** 1 Susan Wakil School of Nursing and Midwifery, Faculty of Medicine and Health, University of Sydney, Sydney, New South Wales, Australia; 2 Institute for Evidence-Based Healthcare, Bond University, Robina, Queensland, Australia; 3 School of Health Sciences, Faculty of Medicine and Health, University of Sydney, Sydney, New South Wales, Australia; 4 The Daffodil Centre, Faculty of Medicine and Health, University of Sydney, Sydney, New South Wales, Australia; 5 Department of Cardiology, Concord Repatriation General Hospital, ANZAC Research Institute, Sydney, New South Wales, Australia

**Keywords:** Cardiac Rehabilitation, Outcome Assessment, Health Care, Global Health

## Abstract

**Aim:**

To systematically evaluate whether relationships between cardiac rehabilitation participation and clinical outcomes, return to work, or knowledge about cardiovascular disease vary across socioeconomic indicators.

**Methods:**

A systematic review was conducted using CENTRAL, CINAHL, Embase and Medline up to 1 November 2024. Studies were included if they compared outcomes between participants who received cardiac rehabilitation and those who did not or received an exercise programme. Outcomes included all-cause death, all-cause and cardiovascular-related rehospitalisation, return to work and cardiovascular knowledge, stratified by socioeconomic indicators. Risk of bias was assessed using the Risk Of Bias In Non-Randomized Studies—of Interventions-I tool.

**Results:**

Six studies involving 555 731 participants were included. Compared with non-participants, cardiac rehabilitation participants had lower rates of all-cause death (12.3%–16.9%) and all-cause rehospitalisation (15.2%–16.1%), with incidence rate differences in cardiovascular-related rehospitalisation reaching up to 27.8 fewer events/100 person-years. Some of the greatest differences were among participants residing in more disadvantaged areas, although this was not consistent across studies. No significant differences were observed in the combined outcome of all-cause death and cardiovascular-related rehospitalisation when stratified by educational attainment levels. Return to work and knowledge outcomes showed greater variation across education and income subgroups, with higher values consistently observed among cardiac rehabilitation participants from less disadvantaged backgrounds. All studies were observational and had moderate risk of bias.

**Conclusions:**

Cardiac rehabilitation improves clinical and functional outcomes across socioeconomic subgroups, although disparities in participation and outcomes persist. Tailoring programme delivery to be more flexible and responsive to literacy needs may help ensure its benefits are equitably achieved across patient subgroups.

**PROSPERO registration number:**

CRD42022332355

**WHAT IS ALREADY KNOWN ON THIS TOPIC**

- People who participated in cardiac rehabilitation were less likely to die or be readmitted to hospital, regardless of their income or education. In some studies, these benefits were even greater among people living in lower income areas.

**WHAT THIS STUDY ADDS**

- We reviewed published studies to understand whether people from different socioeconomic backgrounds get the same benefits from participating in a cardiac rehabilitation programme after a heart attack or other major heart condition.

**HOW THIS STUDY MIGHT AFFECT RESEARCH, PRACTICE OR POLICY**

- Among those who participated in cardiac rehabilitation, people with higher income or education were more likely to return to work and score higher on tests about heart health.

## Introduction

 Cardiovascular disease remains a major contributor to the global health burden. It accounts for one-third of premature deaths and 15% of total healthcare expenditure annually.^1 2^ The burden of cardiovascular disease is not equally distributed and tends to be higher among patients from disadvantaged socioeconomic backgrounds, regardless of area of residence, educational attainment, income level or stage of care.^3^ Barriers such as limited access to public physical activity facilities, less leisure time and lower discretionary income may contribute to the accumulation of cardiovascular risk factors among patients who are from disadvantaged socioeconomic backgrounds.^3^,^6^ A study involving 20 countries reported consistent socioeconomic disparity in cardiovascular outcomes, with more pronounced effects in low-income settings.^7^ In high-income countries, those with secondary or lower education had a 1.2-fold higher risk of recurrent major cardiovascular events and a 1.5-fold higher risk of death compared with those with higher education.^7^ In low-income countries, these risks increased to 1.5-fold and 2.8-fold, respectively.^7^ Similarly, in one high-income setting, patients residing in the most disadvantaged areas had 1.6 to 1.8 times the risk of myocardial infarction and 1.3 to 1.5 times the risk of cardiovascular-related death compared with those in the least disadvantaged areas.^8^

The benefits of cardiac rehabilitation in improving clinical outcomes, including death, rehospitalisation, quality of life and exercise capacity, are well established.^9^,^11^ However, patients with lower educational attainment and less disposable income—who may also have greater long-term care needs—are approximately half as likely to participate in or complete cardiac rehabilitation.^12^ Socioeconomic disparities may not only affect access to cardiac rehabilitation but also influence its delivery and outcomes achieved.^13 14^ While these disparities in access are well documented, to our knowledge, existing studies that report outcomes stratified by socioeconomic subgroups have yet to be systematically synthesised.^15 16^ A systematic review is needed to clarify whether disparities persist in outcomes even when access is achieved, and whether specific strategies are needed to ensure that the benefits of cardiac rehabilitation are equitably distributed across patient populations.

Past reviews have primarily focused on disparities in participation and completion of cardiac rehabilitation, with limited attention to whether its benefits differ once participation is achieved. To our knowledge, no review has yet summarised the extent of these disparities by socioeconomic indicators, including area of residence, educational attainment and income level. This review, therefore, aimed to systematically evaluate whether the relationship between cardiac rehabilitation participation and clinical outcomes, return to work, or knowledge about cardiovascular disease varies across socioeconomic indicators.

## METHODS

This systematic review was conducted according to the protocol registered in the International Prospective Register of Systematic Reviews (PROSPERO; ID: CRD42022332355) and reported adhering to the Preferred Reporting Items for Systematic Reviews and Meta-Analyses (PRISMA) 2020 guidelines.^17^

### Search strategy

A search was performed across four databases: (1) Cochrane Central Register of Controlled Trials (CENTRAL), (2) Cumulative Index of Nursing and Allied Health Literature (CINAHL), (3) Embase and (4) Medline until 1 November 2024. The search strategy included terms related and synonymous to ‘cardiovascular disease’, ‘cardiac rehabilitation’, ‘participation’ and ‘socioeconomic indicator’, using Boolean operators to ensure comprehensive retrieval of studies. The final search strategy is detailed in online supplemental table 1. The database selection and search terms were finalised in collaboration with University of Sydney librarians.

### Study selection

Studies were included if they met the following criteria: (1) the population comprised adults (≥18 years) eligible for cardiac rehabilitation; (2) the intervention was cardiac rehabilitation; (3) the comparator was no cardiac rehabilitation or exercise training programme and (4) the outcome included patients’ death, rehospitalisation, return to work or knowledge about cardiovascular disease, stratified by socioeconomic indicators. Cardiac rehabilitation was defined as a programme that included a baseline assessment of patients’ demographics and clinical characteristics, structured exercise training—whether supervised or unsupervised—and at least one additional component to address cardiovascular risk factors.^18^

Studies were included if published in English or if they had English-language metadata (title, abstract or author-assigned keywords). Non-English studies were translated into English using Google Translate, which has been reported to be sufficiently accurate for selecting studies and extracting data in systematic reviews.^19^ No restrictions were applied on publication period to support comprehensive retrieval of studies.

Titles and abstracts of eligible studies were independently screened by two of three reviewers (DM, QT and AYCJ). Full texts of the potentially relevant studies were again independently screened by two of five reviewers (DM, QT, AYCJ, NSH and DC). Disagreements were resolved through discussion with another reviewer (KH).

### Data extraction

Data from the included studies were independently extracted and tabulated by two of three reviewers (DM, NSH and DC) using a standardised electronic data extraction form. The extracted data included first author, year of publication, country of patient enrolment and study design (number of enrolment centres, enrolment start and end dates and length of follow-up). For both cardiac rehabilitation and comparator groups (no cardiac rehabilitation or exercise training programme), the extracted data included intervention length and setting (face-to-face vs virtual; clinic vs public vs private). For the patient population of each study, the extracted data included baseline demographics (socioeconomic indicators, age and sex), clinical characteristics (comorbidities) and outcomes (all-cause death; all-cause and cardiovascular-related rehospitalisation; return to work; and knowledge about cardiovascular disease). Socioeconomic indicators were extracted as defined and classified in each study, but categorised into area of residence, educational attainment or income level. Definitions were retained and reported as in the original studies in table 1 and compared narratively because national-based socioeconomic indices, such as the Area Deprivation Index in the USA, census-based measures in the Netherlands and Denmark, or income-based measures in Brazil, were conceptually different and harmonising them risked misclassification.^20^,^23^

**Table 1:** Characteristics of the included studies, their cardiac rehabilitation interventions, exercise training program comparator and participants

Characteristic	Eijsvogels *et al*^25^		Guhl *et al*^26^		Kjesbu *et al*^27^		Ghisi *et al*^30^		Pedersen *et al*^28^		Thompson *et al*^29^	
Country	Netherlands		USA		Denmark		Brazil		Denmark		USA	
Study design	Observational cohort study		Observational cohort study		Observational cohort study		Cross-sectional comparative study		Observational cohort study		Observational cohort study	
Number of enrolment centres	89		4		Not specified		2		Not specified		Not specified	
Enrolment period	2012–2017		2010–2018		2015–2018		Not specified		2014–2018		2016–2018	
Length of follow-up (years)	1–7.7		3		0.6–3.3		Not specified		0.25–1		1	
CR vs no CR or exercise training programme	CR	No CR	CR	No CR	CR	No CR	CR	Exercise training programme	CR	No CR	CR	No CR
Number of participants (CR vs no CR)	26 171	57 516	1272	5685	19 383	15 128	42	42	7881	7881	155 872	258 858
Component	6–12 weeks of exercise training and educational sessions (mental health and stress relief, social health and cardiovascular risk management)	Not applicable	Not specified	Not applicable	Immediate phase 1, 8–12 weeks of phase 2 exercise training and educational sessions, and phase 3 follow-up	Not applicable	3–5 times a week of exercise training and educational sessions (dietary counselling, nurse and physician counselling, and psychological guidance)	3 times a week exercise training	2–15 weeks following PCI, or 6–19 following CABG of exercise training and educational sessions	Not applicable	36 sessions of exercise training and educational sessions	Not applicable
Setting	Face-to-face	Not applicable	Face-to-face and/or online	Not applicable	Face-to-face	Not applicable	Face-to-face	Face-to-face	Face-to-face	Not applicable	Face-to-face	Not applicable
Patient population characteristics												
Socioeconomic status	**Socioeconomic status of area:** Least disadvantaged tertile: Upper 30% based on income, wealth, education and recent labour from Statistics Netherlands, Middle tertile: Middle 30%, Most disadvantaged te: Bottom 40%		**Socioeconomic status of area:** Least disadvantaged quartile: Based on poverty, education, housing and employment from ADI by UW, Second least disadvantaged quartile, Second most disadvantaged quartile, Most disadvantaged quartile		**Education:** >3 years of higher education, ≤3 years of higher education, Secondary or vocational education, Primary education		**Education:** Postgraduate studies, Higher education, Incomplete higher education, Secondary education, Primary education, **Income:** >20 times the minimum wage, 10–20 times the minimum wage, 5–10 times the minimum wage, 1–5 times the minimum wage		**Education:** >3 years of higher education, ≤3 years of higher education, Secondary education, Vocational education **Income:** Above the third quartile, Between the first and third quartile		**Socioeconomic status of area:** Least disadvantaged quintile: Based on zip code level rate of education, employment, household income, housing vacancy, local establishments and poverty from Distressed Community Index by the Economic Innovation group, Second least disadvantaged quintile, Middle quintile, Second most disadvantaged quintile, Most disadvantaged quintile	
Mean age (SD) (years)	67 (12)		69 (13)		67 (11)		67 (10)		Age 18–39: 3.2% Age 40–49: 21% Age 50–59: 47% Age 60–70: 29%		75 (7)	
Male (n (**%**))	50 512 (60)		4303 (62)		22 526 (65)		67 (80)		13 738 (87)		269 218 (65)	
Comorbidities	Cancer, dementia, diabetes mellitus, gout, Parkinson's disease, respiratory diseases, thyroid diseases		Atrial fibrillation, coronary artery disease, diabetes, hypertension, liver disease, obesity, stroke		Cancer, chronic obstructive pulmonary disease, depression, kidney disease, muscle or skeletal disease, peripheral artery disease, stroke		Not specified		Anxiety, atrial fibrillation, depression, diabetes, heart failure		Not specified	
Outcome	All-cause death		All-cause death, CVD-related rehospitalisation		All-cause death and CVD-related rehospitalisation		Knowledge about CVD		Return to work		All-cause death, All-cause rehospitalisation, CVD-related rehospitalisation	
Timing of outcome	Date of death		At 36-month follow-up		At 12-month follow-up		Not specified		At 3-, 6-, 9- and 12-month follow-up		At 12-month follow-up	

CR, cardiac rehabilitation; PCI, percutaneous coronary intervention; CABG, coronary artery bypass graft; ADI, Area Deprivation Index; DCI, Distressed Community Index; UW, University of Wisconsin; CVD, cardiovascular disease

### Analysis

The pooled mean and SD of age and the proportion of male participants across all included studies were calculated where available. If the mean age of the intervention or comparator group was not available, or if the mean age of all participants could not be calculated, the proportion of participants across different age subgroups was presented instead.

Outcomes (all-cause death; all-cause and cardiovascular-related rehospitalisation; return to work; and knowledge about cardiovascular disease) were compared between participants who did and did not participate in cardiac rehabilitation, stratified by socioeconomic indicators (area of residence, educational attainment or income level). For area of residence measures based on tertiles, quartiles or quintiles, lower category numbers indicate higher socioeconomic status, while higher numbers indicate lower socioeconomic status. The findings were summarised using narrative synthesis rather than meta-analysis due to differences in study design, outcome measurement, follow-up timing and reporting formats across studies. Results were reported using absolute differences for all-cause death and hospitalisation outcomes, expressed as percentages and incidence rates per 100 person-years; relative differences, expressed as ORs with 95% CIs for return to work and as HRs for the combined outcome of all-cause death and cardiovascular-related rehospitalisation; and mean scores for knowledge about cardiovascular disease.

### Risk of bias assessment

**Figure 1:**
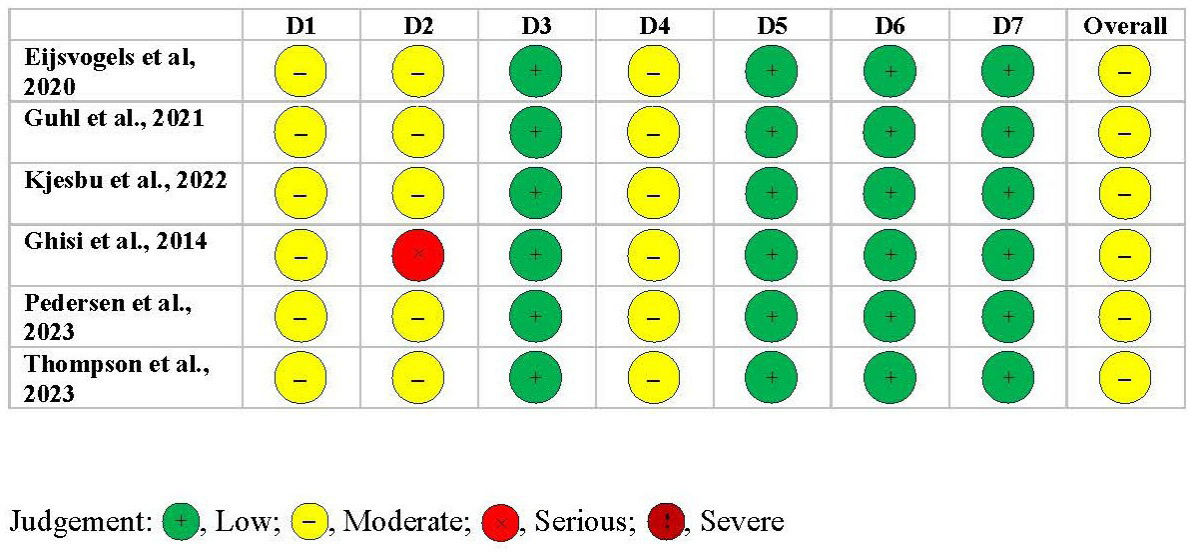
The summary of the risk of bias for each included study.

The quality of the included studies was assessed using the Risk Of Bias In Non-Randomized Studies—of Interventions tool.^24^ This tool assessed bias across seven domains: (1) confounding, (2) selection of participants into the study, (3) classification of interventions, (4) deviations from intended interventions, (5) missing data, (6) measurement of outcomes and (7) selection of the reported result.^24^ The risk of bias of each study was independently assessed by two of four reviewers (DM, NSH, DC and KH), with disagreements resolved through discussion. Summary and graphical results are shown in figures1  2.

**Figure 2:**
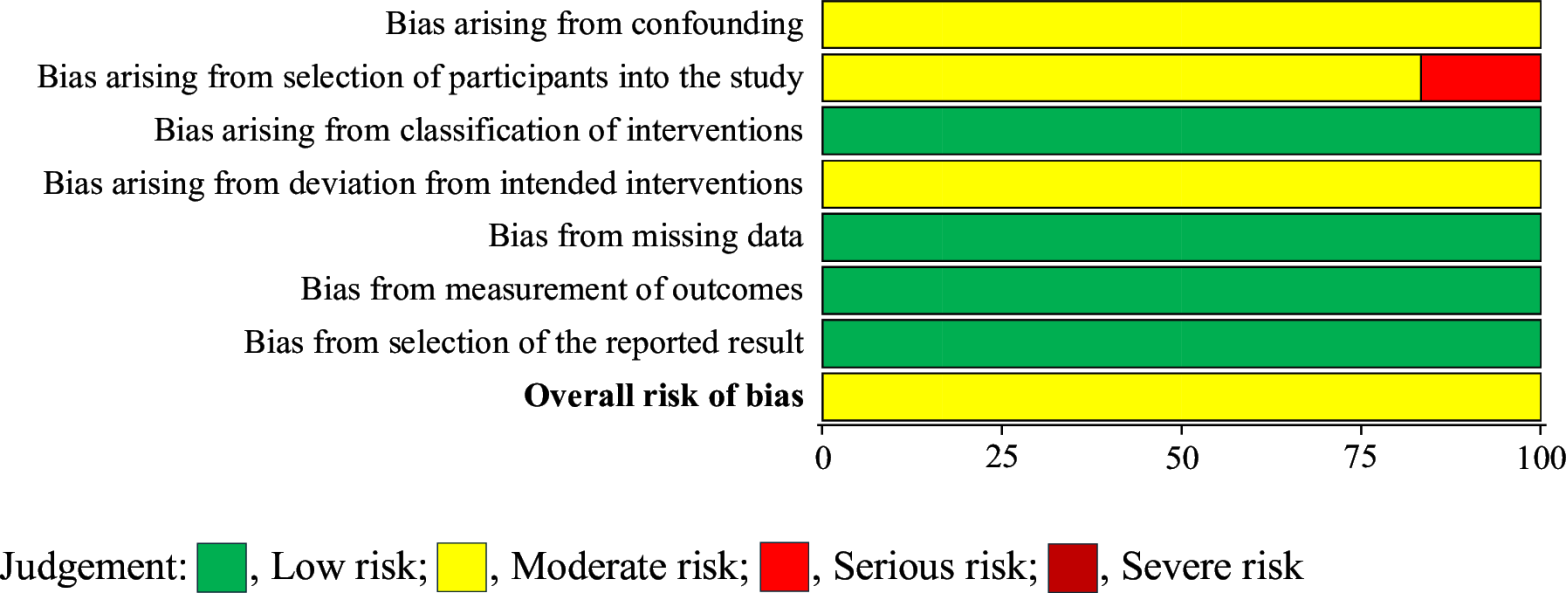
The graph of the risk of bias for all included studies.

## Results

### Study selection

**Figure 3:**
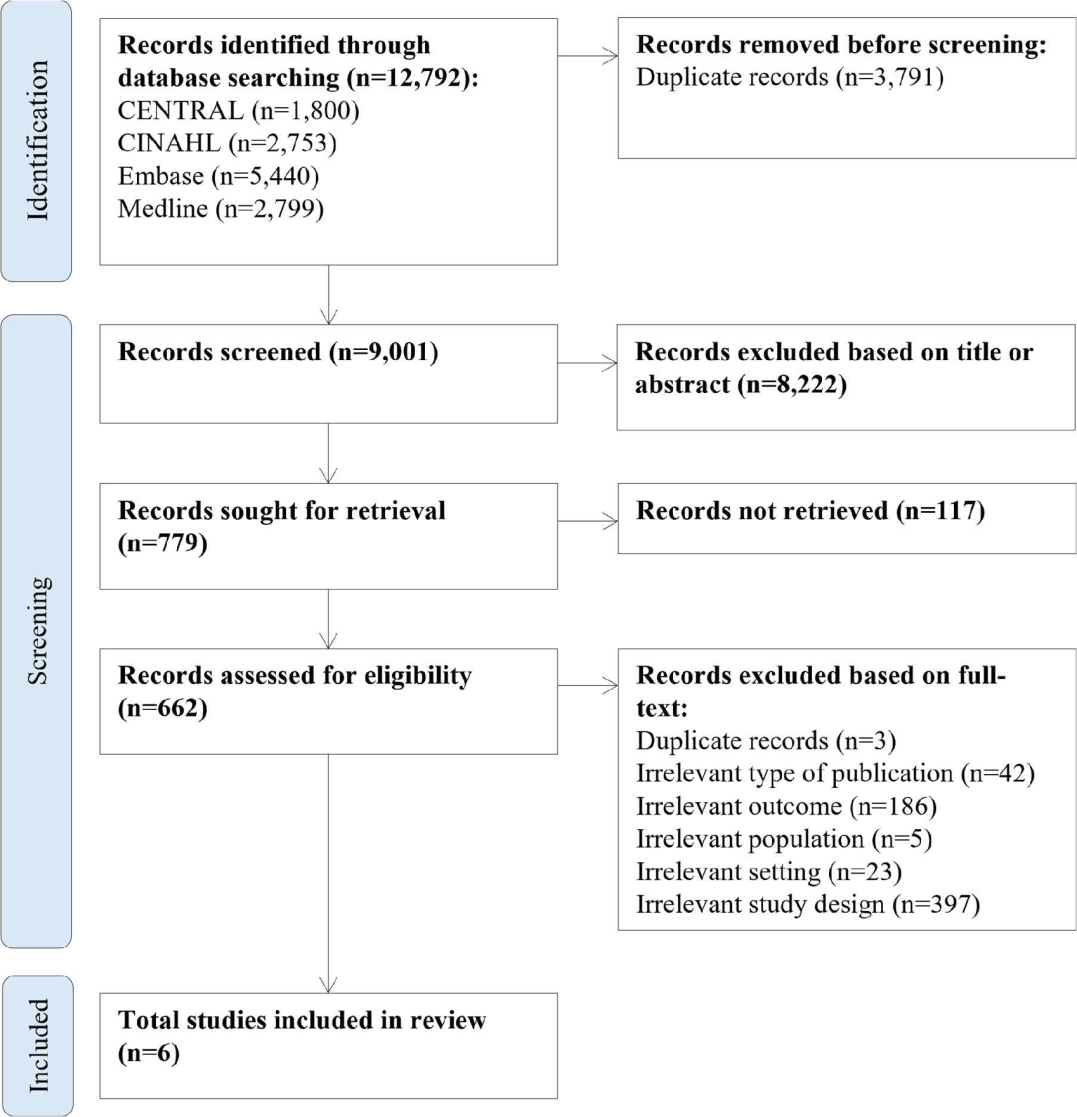
The Preferred Reporting Items for Systematic reviews and Meta-Analyses (PRISMA) flow diagram of the study selection process.

The systematic review was conducted and reported in accordance with the PRISMA 2020 guidelines. A total of 7756 non-duplicated studies were screened. Six studies—five observational cohort studies and one cross-sectional comparative study—were included, comprising 555 731 participants (figure 3).^25^,^30^ The characteristics of the included studies, their cardiac rehabilitation interventions, exercise training programme comparator and patient populations are summarised in table 1.

The included studies were conducted in the Netherlands, USA, Denmark and Brazil, across at least 95 centres. Three studies referred to multiple centres without specifying exact numbers. Follow-up periods ranged from 3 months to 8 years (table 1). Five studies compared cardiac rehabilitation (n=2 10 579) with no cardiac rehabilitation (n=3 45 068).^25^,^29^ One study compared cardiac rehabilitation (n=42) with an exercise training programme (n=42).^30^ The mean age across five of the six studies was 73 (SD: 8) years. Sixty-five per cent (360 364/555 731) of participants across the six studies were male.

### All-cause death

All-cause death was evaluated in three observational studies, with follow-up periods ranging from 1 to 8 years.^25 26 29^ Outcomes were reported either as numbers and percentages or incidence rates (per 100 person-years), stratified by cardiac rehabilitation participation and socioeconomic status of area of residence. Across studies, cardiac rehabilitation participants (n=173 315) had lower rates of all-cause death compared with those who did not participate (n=322 060), with differences ranging from 12.3% to 16.9%, depending on socioeconomic subgroups (table 2).

**Table 2:** Outcomes of the included studies, stratified by cardiac rehabilitation participation and socioeconomic indicator (defined by area of residence, education or income)

Outcome	Study	Socioeconomic indicator	Socioeconomic subgroups*	CR	No CR or exercise training programme	Difference
All-cause death	Eijsvogels *et al*^25^	Socioeconomic status of area	Tertile 1	n (%): 444 (6.6)	n (%): 2974 (20.7)	−14.1%
			Tertile 2	n (%): 622 (7.6)	n (%): 4086 (23.3)	−15.7%
			Tertile 3	n (%): 900 (8.0)	n (%): 6383 (24.9)	−16.9%
	Guhl *et al*^26^	Socioeconomic status of area	Quartile 1	Incidence rate (per 100 person-year): 1.9 (95% CI: 1.0 to 3.5)	Incidence rate (per 100 person-year): 17.0 (95% CI: 14.8 to 19.6)	Incidence rate (per 100 person-year): −15.1
			Quartile 2	Incidence rate (per 100 person-year): 2.7 (95% CI: 1.8 to 3.8)	Incidence rate (per 100 person-year): 14.1 (95% CI: 12.9 to 14.4)	Incidence rate (per 100 person-year): −11.4
			Quartile 3	Incidence rate (per 100 person-year): 2.9 (95% CI: 2.1 to 4.0)	Incidence rate (per 100 person-year): 15.3 (95% CI: 14.3 to 16.5)	Incidence rate (per 100 person-year): −12.4
			Quartile 4	Incidence rate (per 100 person-year): 1.8 (95% CI: 1.1 to 3.0)	Incidence rate (per 100 person-year): 15.4 (95% CI: 14.2 to 16.7)	Incidence rate (per 100 person-year): −13.6
	Thompson *et al*^29^	Socioeconomic status of area	Quintile 1	n (%): 899 (2.0)	n (%): 7632 (14.6)	−12.6%
			Quintile 2	n (%): 857 (2.3)	n (%): 7662 (14.7)	−12.4%
			Quintile 3	n (%): 770 (2.5)	n (%): 7531 (14.8)	−12.3%
			Quintile 4	n (%): 655 (2.6)	n (%): 8007 (15.1)	−12.5%
			Quintile 5	n (%): 472 (2.7)	n (%): 7746 (15.4)	−12.7%
All-cause rehospitalisation	Thompson *et al*^29^	Socioeconomic status of area	Quintile 1	n (%): 13 979 (31.3)	n (%): 24 551 (47.0)	−15.7%
			Quintile 2	n (%): 11 728 (31.8)	n (%): 25 070 (47.9)	−16.1%
			Quintile 3	n (%): 10 019 (32.3)	n (%): 24 234 (47.5)	−15.2%
			Quintile 4	n (%): 8472 (33.0)	n (%): 25 663 (48.5)	−15.5%
			Quintile 5	n (%): 5094 (33.4)	n (%): 24 673 (49.0)	−15.6%
CVD-related rehospitalisation	Guhl *et al*^26^	Socioeconomic status of area	Quartile 1	Incidence rate (per 100 person-year): 19.3 (95% CI: 15.4 to 24.3)	Incidence rate (per 100 person-year): 42.6 (95% CI: 38.1 to 47.7)	Incidence rate (per 100 person-year): −23.3
			Quartile 2	Incidence rate (per 100 person-year): 17.7 (95% CI: 15.0 to 20.8)	Incidence rate (per 100 person-year): 41.4 (95% CI: 38.7 to 44.3)	Incidence rate (per 100 person-year): −23.7
			Quartile 3	Incidence rate (per 100 person-year): 19.5 (95% CI: 16.8 to 22.6)	Incidence rate (per 100 person-year): 42.0 (95% CI: 39.8 to 44.4)	Incidence rate (per 100 person-year): −22.5
			Quartile 4	Incidence rate (per 100 person-year): 19.3 (95% CI: 15.9 to 23.3)	Incidence rate (per 100 person-year): 47.1 (95% CI: 44.3 to 50.1)	Incidence rate (per 100 person-year): −27.8
	Thompson *et al*^29^	Socioeconomic status of area	Quintile 1	n (%): 2481 (5.6)	n (%): 5594 (10.7)	−5.1%
			Quintile 2	n (%): 2199 (6.0)	n (%): 5605 (10.7)	−4.7%
			Quintile 3	n (%): 1997 (6.4)	n (%): 5499 (10.8)	−4.4%
			Quintile 4	n (%): 1695 (6.6)	n (%): 6164 (11.6)	−5.0%
			Quintile 5	n (%): 1176 (6.7)	n (%): 5955 (11.8)	−5.1%
All-cause death and CVD-related rehospitalisation	Kjesbu *et al*^27^	Education attainment	>3 years of higher education			HR: 1.20 (95% CI: 0.89 to 1.50)†
			≤3 years of higher education			HR: 0.96 (95% CI: 0.83 to 1.10)†
			Secondary or vocational education			HR: 1.00 (95% CI: 0.94 to 1.10)†
			Primary education			HR: 1.00 (95% CI: 0.94 to 1.10)†
Return to work	Pedersen *et al*.^28^	Education attainment	>3 years of higher education			OR at 3-month: 1.35 (95% CI: 1.21 to 1.51)
						OR at 6-month: 1.16 (95% CI: 1.02 to 1.33)
						OR at 9-month: 1.26 (95% CI: 1.09 to 1.46)
						OR at 1-year: 1.23 (95% CI: 1.06 to 1.44)
			≤3 years of higher education			OR at 3-month: 1.09 (95% CI: 1.01 to 1.18)
						OR at 6-month: 1.09 (95% CI: 0.99 to 1.20)
						OR at 9-month: 1.29 (95% CI: 1.16 to 1.43)
						OR at 1-year: 1.33 (95% CI: 1.19 to 1.48)
			Secondary education			OR at 3-month: 1.28 (95% CI: 1.09 to 1.51)
						OR at 6-month: 1.35 (95% CI: 1.10 to 1.66)
						OR at 9-month: 1.42 (95% CI: 1.13 to 1.78)
						OR at 1-year: 1.36 (95% CI: 1.08 to 1.71)
			Vocational education			OR at 3-month: 0.98 (95% CI: 0.92 to 1.04)
						OR at 6-month: 0.98 (95% CI: 0.92 to 1.05)
						OR at 9-month: 1.08 (95% CI: 1.01 to 1.17)
						OR at 1-year: 1.10 (95% CI: 1.02 to 1.19)
	Pedersen *et al*^28^	Socioeconomic status of area	Quartile 1			OR at 3-month: 1.90 (95% CI: 1.74 to 2.07)
						OR at 6-month: 2.36 (95% CI: 2.14 to 2.61)
						OR at 9-month: 2.31 (95% CI: 2.07 to 2.57)
						OR at 1-year: 2.41 (95% CI: 2.15 to 2.69)
			Quartile 2–4			OR at 3-month: 1.28 (95% CI: 1.19 to 1.38)
						OR at 6-month: 1.62 (95% CI: 1.50 to 1.76)
						OR at 9-month: 1.61 (95% CI: 1.48 to 1.76)
						OR at 1-year: 1.57 (95% CI: 1.44 to 1.72)
Knowledge about CVD	Ghisi et al., 2014	Education attainment	Postgraduate studies	Mean score: 46.0 (SD: 2.0)	Mean score: 41.3 (SD: 6.6)	Difference in mean score: 4.7
			Higher education	Mean score: 45.9 (SD: 4.8)	Mean score: 40.0 (SD: 7.3)	Difference in mean score: 5.9
			Incomplete higher education	Mean score: 48.3 (SD: 4.0)	Mean score: 31.0 (SD: 6.0)	Difference in mean score: 17.3
			Secondary education	Mean score: 36.0 (SD: 8.1)	Mean score: 34.3 (SD: 4.7)	Difference in mean score: 1.7
			Primary education	Mean score: 29.5 (SD: 3.5)	Mean score: 27.2 (SD: 8.5)	Difference in mean score: 2.3
	Ghisi et al., 2014	Income	Above 20 times the minimum wage	Mean score: 46.1 (SD: 7.0)	Mean score: 40.0 (SD: 7.5)	Difference in mean score: 6.1
			10–20 times the minimum wage	Mean score: 43.9 (SD: 6.9)	Mean score: 38.1 (SD: 9.9)	Difference in mean score: 5.8
			5–10 times the minimum wage	Mean score: 34.8 (SD: 12.0)	Mean score: 35.3 (SD: 6.3)	Difference in mean score: −0.5
			1–5 times the minimum wage minimum wage	No participants	Mean score: 33.2 (SD: 6.3)	NA

* For tertiles, quartiles and quintiles, lower category numbers indicate higher socioeconomic status, while higher numbers indicate lower socioeconomic status.

† Adjusted for age, sex, comorbidity index, hypertension, diabetes, hypercholesterolaemia and index event.

CR, cardiac rehabilitation; CVD, cardiovascular disease; NA, not applicable.;

Eijsvogels *et al*^25^ reported a progressive increase in difference between cardiac rehabilitation and no cardiac rehabilitation groups across socioeconomic tertiles: from −14.1% in the least disadvantaged tertile, −15.7% in the middle tertile, to −16.9% in the most disadvantaged tertile. Guhl *et al*^26^ reported the greatest incidence rate difference in the least disadvantaged quartile (−15.1 events per 100 person-years), followed by smaller differences in middle quartiles (−11.4 to −12.4 events per 100 person-years), and a modest increase again in the most disadvantaged quartile (−13.6 events per 100 person-years). In contrast, Thompson *et al*^29^ reported comparable differences across all quintiles, with absolute differences ranging from −12.3% to −12.7%.

### Rehospitalisation

#### All-cause rehospitalisation

All-cause rehospitalisation was evaluated in one observational study with a 1-year follow-up.^29^ Outcomes were reported as numbers and percentages, stratified by cardiac rehabilitation participation and socioeconomic status of area of residence. Cardiac rehabilitation participants (n=1 55 872) had lower rates of all-cause rehospitalisation compared with those who did not participate (n=2 58 858). Thompson *et al*^29^ reported comparable differences between cardiac rehabilitation and no cardiac rehabilitation groups across all socioeconomic quintiles, with absolute differences ranging from −15.2% to −16.1%. This was consistent with the comparable differences reported for all-cause death in the same study, which were also observed across all quintiles.

#### Cardiovascular-related rehospitalisation

Cardiovascular-related rehospitalisation was evaluated in two observational studies, with follow-up periods ranging from 1 to 3 years.^26 29^ Outcomes were reported either as numbers and percentages or incidence rates (per 100 person-years), stratified by cardiac rehabilitation participation and socioeconomic status of area of residence. Cardiac rehabilitation participants (n=1 57 144) had lower rates of cardiovascular-related rehospitalisation compared with those who did not participate (n=2 64 543), with differences depending on socioeconomic subgroups (table 2).

Guhl *et al*^26^ reported incidence rate difference between cardiac rehabilitation and no cardiac rehabilitation groups that were relatively comparable across most socioeconomic quartiles, ranging from −22.5 to −23.3 events per 100 person-years. The greatest difference was observed in the most disadvantaged quartile (−27.8 events per 100 person-years). Meanwhile, Thompson *et al*^29^ reported comparable differences across all quintiles, with absolute differences ranging from −4.4% to −5.1%. These were consistent with the comparable differences reported for all-cause death and all-cause rehospitalisation in the same study, which were also observed across all quintiles.

### All-cause death and cardiovascular-related rehospitalisation

The combined outcome of all-cause death and cardiovascular-related rehospitalisation was evaluated in one observational study with a 1-year follow-up.^27^ Outcomes were reported as HRs and corresponding 95% CIs, stratified by cardiac rehabilitation participation and educational attainment (>3 years of higher education, ≤3 years of higher education, secondary or vocational education and primary education). Cardiac rehabilitation participants (n=19 383) had no statistically significant differences in rates of the combined outcome compared with those who did not participate (n=15 128), across socioeconomic subgroups.

While not statistically significant, Kjesbu *et al*^27^ reported the highest HR between cardiac rehabilitation and no cardiac rehabilitation groups among participants whose highest attained education was >3 years of higher education (HR: 1.20, 95% CI 0.89 to 1.50), slightly lower among those whose highest attained education was ≤3 years of higher education (HR: 0.96, 95% CI 0.83 to 1.10), and identical among those whose highest attained education was secondary or vocational education (HR: 1.00, 95% CI 0.94 to 1.10), and primary education (HR: 1.00, 95% CI 0.94 to 1.10).^27^

### Return to work

Return to work was evaluated in one observational study with a 1-year follow-up.^28^ Outcomes were reported as ORs and corresponding 95% CIs at 3, 6, 9 months and 1 year, stratified by cardiac rehabilitation participation and two socioeconomic indicators: educational attainment (>3 years of higher education, ≤3 years of higher education, secondary education and vocational education) and income level (above the third quartile and between the first and third quartile). Cardiac rehabilitation participants (n=7881) had higher odds of returning to work compared with those who did not participate (n=7881), with differences depending on both education and income subgroups (table 2).

Pedersen *et al*^28^ reported that differences in return to work between cardiac rehabilitation and no cardiac rehabilitation groups were observed at all time points among participants whose highest attained education was secondary education or higher education. Among those whose highest attained education was vocational education, no differences were observed at 3 and 6 months, but higher odds were observed at 9 months (OR: 1.08, 95% CI 1.01 to 1.17) and 12 months (OR: 1.10, 95% CI 1.02 to 1.19). At 1 year, the highest odds were observed among those whose highest attained education was secondary education (OR: 1.36, 95% CI 1.08 to 1.71) and ≤3 years of higher education, followed by >3 years of higher education (OR: 1.23, 95% CI 1.06 to 1.44) and vocational education (OR: 1.10, 95% CI 1.02 to 1.19).

When stratified by income, Pedersen *et al*^28^ reported participants with incomes above the third quartile had the greatest odds of returning to work at 1 year (1.23, 95% CI 1.06 to 1.44), followed by those with incomes between the first and third quartiles (OR: 1.10, 95% CI 1.02 to 1.19).

### Knowledge about cardiovascular disease

Knowledge about cardiovascular disease was evaluated in one cross-sectional comparative study.^30^ Outcomes were reported as mean knowledge scores, stratified by cardiac rehabilitation participation and two socioeconomic indicators: educational attainment (postgraduate studies, higher education, incomplete higher education, secondary education and primary education) and income level (>20 times the minimum wage, 10–20 times the minimum wage, 5–10 times the minimum wage and 1 to 5 times the minimum wage). Cardiac rehabilitation participants (n=48) had higher knowledge scores compared with those who received an exercise training programme without an educational session (n=48), with differences depending on both education and income subgroups (table 2).

Ghisi *et al*^30^ reported higher knowledge scores between cardiac rehabilitation and no cardiac rehabilitation groups among participants with higher educational attainment. The greatest differences were observed among participants whose highest attained education was incomplete higher education (+17.3 points), followed by those whose highest attained education was postgraduate studies (+4.7 points), higher education (+5.9 points), secondary education (+1.7 points) and primary education (+2.3 points).

When stratified by income level, similar trends were observed. The greatest differences were observed among participants who earned >20 times the minimum wage (+6.1 points), followed by those who earned 10–20 times the minimum wage (+5.8 points). In contrast, no difference was observed among those who earned 5–10 times the minimum wage and no participants who earned 1 to 5 times the minimum wage received cardiac rehabilitation. However, thresholds for clinical significance were not reported, limiting conclusions whether score differences reflect meaningful improvements.

### Risk of bias assessment

All six included studies were classified as having a moderate risk of bias, primarily related to confounding, selection of participants and deviation from intended interventions (figures1  2). Moderate bias related to confounding was observed in all six included studies, attributed to their observational study designs and limited randomisation. Moderate bias related to selection of participants was observed in five included studies and serious risk was observed in one. This bias was attributed to the under-representation of participants from more disadvantaged socioeconomic backgrounds in the cardiac rehabilitation groups. While moderate bias related to deviation from intended intervention was observed in all six included studies, attributed to the limited reporting on adherence rates to either cardiac rehabilitation or exercise training programme. Low risk was observed in all six included studies for classification of interventions, missing data, measurement of outcomes and selection of the reported result.

## Discussion

The current systematic review evaluated whether the relationship between participation in cardiac rehabilitation and clinical or functional outcomes varied across socioeconomic indicators. Cardiac rehabilitation participation was consistently linked with lower rates of all-cause death, all-cause rehospitalisation and cardiovascular-related rehospitalisation. The overall direction of findings was consistent across studies. Differences between cardiac rehabilitation and no cardiac rehabilitation were observed across socioeconomic subgroups, with some studies reporting greater differences in all-cause death and cardiovascular-related rehospitalisation among participants residing in more disadvantaged areas. However, this trend was not consistent across all studies. No significant differences were found in the effect of cardiac rehabilitation on the combined outcome across education levels, with HRs highest in the most educated and lowest in those with primary or vocational education. In contrast, return to work and knowledge about cardiovascular disease outcomes showed greater variation across socioeconomic subgroups. The greatest differences were among participants who attained higher educational levels or earned higher income levels. All six studies showed a moderate risk of bias, primarily in the domains of confounding, selection of participants and deviations from intended interventions, necessitating careful interpretation of findings.

Barriers to accessing cardiac rehabilitation remain a challenge for patients who may benefit the most from participation. Those from disadvantaged socioeconomic backgrounds often face a greater burden of cardiovascular risk factors, experience poorer cardiovascular outcomes, yet have lower participation in cardiac rehabilitation.^31^,^36^ Encouragingly, some of the largest differences in all-cause death and rehospitalisation were observed among participants residing in more disadvantaged areas, suggesting that cardiac rehabilitation may support greater cardiovascular risk reduction in this subgroup.^9^,^1137^ Increasing participation among patients from disadvantaged backgrounds is therefore essential to ensure they can benefit from cardiac rehabilitation.^31^,^36^ Strategies such as expanding access to digital or virtual cardiac rehabilitation programmes may help address logistical barriers, particularly for patients with limited transportation options or less flexible working hours.^38^,^40^ Transport support, such as van rides, public transport vouchers or partnerships with ride-share services, could also help improve access, but would likely require additional external funding.^41^ These strategies could build on previous interventions that have successfully reduced missed healthcare appointments.^41^ Greater integration of general practitioners into the delivery of cardiac rehabilitation components, such as in-person or virtual consultation, exercise prescriptions or individualised educational sessions on risk management, may also support cardiac rehabilitation access by embedding secondary prevention into routine and ongoing contacts with familiar primary care providers.^42 43^

Despite comparable clinical outcomes across educational attainment levels, patients who attained higher education or earned higher income demonstrated greater improvements in return to work and knowledge about cardiovascular disease. This suggests potential disparities in health literacy and self-efficacy, even when cardiac rehabilitation includes educational sessions. Patients who attained higher education or earned higher income may be better equipped to apply learnings from the educational sessions, seek additional resources and support independently, and negotiate flexible transition back to work in a modified capacity.^44^,^46^ To address these disparities, cardiac rehabilitation programmes may benefit from tailoring educational materials to different literacy levels or extending sessions for those needing additional support.^4447^,^49^ Obtaining programme and encouraging providers certification may also help ensure accessibility and consistency of these educational materials.^48 49^ Yet, longitudinal research is still needed to explore how work-related outcomes differ by factors such as employment status (full-time, part-time, modified roles vs retirement) or occupation type (blue-collar vs white-collar) and how these influence education or income.

Beyond the bias within the eligible studies, several limitations remain in the current systematic review. First, in five of the included studies, it could not be ascertained whether participants who did not participate in cardiac rehabilitation also refrained from engaging in structured exercise independently, whether supervised or unsupervised, outside of formal healthcare setting. This limited the ability to fully contrast these groups to the one study that compared cardiac rehabilitation to an exercise-only training programme. Second, heterogeneity in the definitions and categorisation of socioeconomic indicators, as well as inconsistencies in sample size, outcome measurement and timing, limited the comparability and generalisability of the findings. We reported the study by Ghisi *et al*^30^, which contributed fewer than 100 participants and only to the outcome of knowledge about cardiovascular disease, as narrative summary rather than including it in the pooled analysis. Given the variability in definitions and classifications of socioeconomic indicators across countries, we retained and reported them as in the original studies and presented outcome differences stratified by these definitions. This variability likely contributed to the differences observed across studies. Additionally, future studies could evaluate broader outcomes, such as psychosocial recovery, quality of life and contacts with primary care providers, to better reflect the multifaceted benefits of cardiac rehabilitation across populations from varied socioeconomic backgrounds.

## Conclusion

Cardiac rehabilitation supports lower rates of all-cause death and rehospitalisation across socioeconomic subgroups, with some of the greatest differences observed among participants residing in more disadvantaged areas. Participants with higher education or income levels appear more likely to return to work and report greater knowledge gains following participation. These findings highlight disparities in how different socioeconomic subgroups benefit from cardiac rehabilitation. Addressing these disparities may require more tailored and flexible digital or virtual delivery of programmes, including transport support, stronger primary care integration and accessible educational sessions.

## Supplementary material

10.1136/openhrt-2025-003698 online supplemental file 1

## Data Availability

Data are available upon reasonable request.
