# Supplementary material for: Socioeconomic variation in the relationship between cardiac rehabilitation participation and clinical outcomes: a systematic review
Source: Open Heart. 2025 Dec 7;12(2):e003698. doi: 10.1136/openhrt-2025-003698 (PMC12699550; doi:10.1136/openhrt-2025-003698)
Supplement: online supplemental file 1 [file openhrt-12-2-s001.docx]

# **Supplementary Material**

## Supplementary Table 1. Final search terms related and synonym to ‘cardiovascular disease’ eligible for cardiac rehabilitation, ‘cardiac rehabilitation’, ‘participation’ and ‘socioeconomic indicator’

| **CENTRAL** | **CINAHL** | **Embase** | **Medline** |
| --- | --- | --- | --- |
| 1. Acute Coronary Syndrome/ 2. Angina Pectoris/ 3. angina*.mp. 4. angioplast*.mp. 5. Atrial Fibrillation/ 6. atrial fibrillation*.mp. 7. atherectom*.mp. 8. "*cardia* arrest".mp. 9. Cardiomyopathies/ 10. cardiomyopath*.mp. 11. Myocardial Revascularization/ 12. Coronary Artery Disease/ 13. Coronary Thrombosis/ 14. (coronary adj3 (bypass or disease* or dilat* or interven* or revascular* or syndrome* or thrombo*)).mp. 15. Defibrillators/ 16. defibrillat*.mp. 17. endoluminal repair*.mp. 18. Heart Arrest/ 19. Heart Failure/ 20. Heart Transplantation/ 21. (heart adj3 (attack* or disease* or fail* or infarct* or interven* or isch?emi* or transplant*)).mp. 22. Hypercholesterolemia/ 23. hypercholesterol?emia*.mp. 24. Myocardial Infarction/ 25. (myocardi* adj3 (infarct* or isch?emi*)).mp. 26. Pacemaker, Artificial/ 27. pacemaker*.mp. 28. Percutaneous Coronary Intervention/ 29. percutaneous coronary intervention*.mp. 30. Stents/ 31. stent*.mp. 32. Heart Valve Prosthesis Implantation/ 33. (valve* adj3 (device* or implant* or repair* or replace* or surger*)).mp. 34. (vascular adj3 (disease* or interven*)).mp. 35. (ventricular adj3 device*).mp. 36. "*stemi".mp. 37. percutaneous transluminal coronary angioplast*.mp. 38. 1 or 2 or 3 or 4 or 5 or 6 or 7 or 8 or 9 or 10 or 11 or 12 or 13 or 14 or 15 or 16 or 17 or 18 or 19 or 20 or 21 or 22 or 23 or 24 or 25 or 26 or 27 or 28 or 29 or 30 or 31 or 32 or 33 or 34 or 35 or 36 or 37 | 1. (MH “Angina Pectoris”) 2. “angina*” 3. (MH “Angioplasty”) 4. “angioplast*” 5. (MH “Atrial Fibrillation”) 6. “atrial fibrillation*” 7. “atherectom*” 8. (MH “Heart Arrest”) 9. “*cardia* arrest” 10. (MH “Cardiomyopathies, Alcoholic”) OR (MH “Cardiomyopathy, Dilated”) OR (MH “Cardiomyopathy, Hypertrophic”) 11. “cardiomyopath*” 12. (MH “Myocardial Revascularization”) 13. (MH “Myocardial Ischemia”) 14. (MH “Coronary Thrombosis”) 15. (coronary W3 (bypass or disease* or dilat* or interven* or revascular* or syndrome* or thrombo*)) 16. (MH “Defibrillators”) 17. “defibrillat*” 18. “endoluminal repair*” 19. (MH “Heart Failure”) 20. (MH “Heart Transplantation”) 21. (heart W3 (attack* or disease* or fail* or infarct* or interven* or isch?emi* or transplant*) 22. (MH “Hypercholesterolemia”) 23. “hypercholesterol#emia*” 24. (myocardi* W3 (infarct* or isch#emi*)) 25. (MH “Pacemaker, Artificial”) 26. “pacemaker*” 27. (MH “Percutaneous Coronary Intervention”) 28. “percutaneous coronary intervention*” 29. (MH “Stents”) 30. “stent*” 31. (MH “Heart Valve Prosthesis”) 32. (valve* W3 (device* or implant* or repair* or replace* or surger*)) 33. (vascular W3 (disease* or interven*)) 34. (ventricular W3 device*) 35. “percutaneous transluminal coronary angioplast*” 36. (MH “ST Elevation Myocardial Infarction”) 37. "*stemi" 38. S1 OR S2 OR S3 OR S4 OR S5 OR S6 OR S7 OR S8 OR S9 OR S10 OR S11 OR S12 OR S13 OR S14 OR S15 OR S16 OR S17 OR S18 OR S19 OR S20 OR S21 OR S22 OR S23 OR S24 OR S25 OR S26 OR S27 OR S28 OR S29 OR S30 OR S31 OR S32 OR S33 OR S34 OR S35 OR S36 OR S37 | 1. angina pectoris/ 2. angina*.mp. 3. angioplasty/ 4. angioplast*.mp. 5. atrial fibrillation/ 6. atrial fibrillation*.mp. 7. "*cardia* arrest".mp. 8. cardiomyopathy/ 9. cardiomyopath*.mp. 10. coronary artery bypass graft/ 11. coronary artery disease/ 12. (coronary adj3 (bypass or disease* or dilat* or interven* or revascular* or syndrome* or thrombo*)).mp. 13. defibrillat*.mp. 14. endoluminal repair*.mp. 15. heart failure/ 16. heart transplantation/ 17. (heart adj3 (attack* or disease* or fail* or infarct* or interven* or isch?emi* or transplant*)).mp. 18. hypercholesterolemia/ 19. hypercholesterol?emia*.mp. 20. heart infarction/ 21. (myocardi* adj3 (infarct* or isch?emi*)).mp. 22. cardiac rhythm management device/ 23. pacemaker*.mp. 24. percutaneous coronary intervention/ 25. percutaneous coronary intervention*.mp. 26. cardiovascular stent/ 27. stent*.mp. 28. transcatheter aortic valve implantation/ 29. (valve* adj3 (device* or implant* or repair* or replace* or surger*)).mp. 30. (vascular adj3 (disease* or interven*)).mp. 31. (ventricular adj3 device*).mp. 32. "*stemi".mp. 33. percutaneous transluminal coronary angioplast*.mp. 34. atherectom*.mp. 35. 1 or 2 or 3 or 4 or 5 or 6 or 7 or 8 or 9 or 10 or 11 or 12 or 13 or 14 or 15 or 16 or 17 or 18 or 19 or 20 or 21 or 22 or 23 or 24 or 25 or 26 or 27 or 28 or 29 or 30 or 31 or 32 or 33 or 34 | 1. Angina Pectoris/ 2. angina*.mp. 3. angioplast*.mp. 4. Atrial Fibrillation/ 5. atrial fibrillation*.mp. 6. atherectom*.mp. 7. "*cardia* arrest".mp. 8. Cardiomyopathies/ 9. cardiomyopath*.mp. 10. Coronary Artery Bypass/ 11. Coronary Artery Disease/ 12. Acute Coronary Syndrome/ 13. Coronary Thrombosis/ 14. (coronary adj3 (bypass or disease* or dilat* or interven* or revascular* or syndrome* or thrombo*)).mp. 15. Defibrillators, Implantable/ 16. defibrillat*.mp. 17. endoluminal repair*.mp. 18. Heart Arrest/ 19. Heart Failure/ 20. Heart Transplantation/ 21. (heart adj3 (attack* or disease* or fail* or infarct* or interven* or isch?emi* or transplant*)).mp. 22. Hypercholesterolemia/ 23. hypercholesterol?emia*.mp. 24. Myocardial Infarction/ 25. (myocardi* adj3 (infarct* or isch?emi*)).mp. 26. Pacemaker, Artificial/ 27. pacemaker*.mp. 28. Percutaneous Coronary Intervention/ 29. percutaneous coronary intervention*.mp. 30. Stents/ 31. stent*.mp. 32. Heart Valve Prosthesis Implantation/ 33. (valve* adj3 (device* or implant* or repair* or replace* or surger*)).mp. 34. (vascular adj3 (disease* or interven*)).mp. 35. (ventricular adj3 device*).mp. 36. "*stemi".mp. 37. percutaneous transluminal coronary angioplast*.mp. 38. 1 or 2 or 3 or 4 or 5 or 6 or 7 or 8 or 9 or 10 or 11 or 12 or 13 or 14 or 15 or 16 or 17 or 18 or 19 or 20 or 21 or 22 or 23 or 24 or 25 or 26 or 27 or 28 or 29 or 30 or 31 or 32 or 33 or 34 or 35 or 36 or 37 |
| 1. exp Cardiac Rehabilitation/ 2. (cardi* adj3 rehab*).mp. 3. heart adj3 rehab*.mp. 4. Exercise Therapy/ 5. ((aerobic or balance or behavio?r* or endurance or exercise* or fitness* or flexibility* or physical or resistan* or strength) adj3 (activit* or intervention* or therap* or training* or treatment* or prescription* or program*)).tw, kw. 6. 39 or 40 or 41 or 42 or 43 | 1. (MH “Rehabilitation, Cardiac+”) 2. (MH “American Association of Cardiovascular and Pulmonary Rehabilitation”) OR (MH “Cardiac Rehabilitation (Saba CCC)”) OR (MH “Cardiac Care: Rehabilitative (Iowa NIC)”) 3. (cardi* W3 rehab*) 4. (heart W3 rehab*) 5. (MH “Physical Therapy”) 6. ((aerobic or balance or behavio#r* or endurance or exercise* or fitness* or flexibility* or physical or resistan* or strength) W3 (activit* or intervention* or therap* or training* or treatment* or prescription* or program*)) 7. S39 OR S40 OR S41 OR S42 OR S43 OR S44 | 1. exp heart rehabilitation/ 2. (cardi* adj3 rehab*).mp. 3. (heart adj3 rehab*).mp. 4. physical medicine/ 5. ((aerobic or balance or behavio?r* or endurance or exercise* or fitness* or flexibility* or physical or resistan* or strength) adj3 (activit* or intervention* or therap* or training* or treatment* or prescription* or program*)).tw, kw. 6. 36 or 37 or 38 or 39 or 40 | 1. Cardiac Rehabilitation/ 2. (cardi* adj3 rehab*).mp. 3. heart adj3 rehab*).mp. 4. Exercise Therapy/ 5. ((aerobic or balance or behavio?r* or endurance or exercis* or fitness* or flexibility* or physical or resistan* or strength) adj3 (activit* or intervention* or therap* or training* or treatment* or prescription* or program*)).tw, kw. 6. 39 or 40 or 41 or 42 or 43 |
| 1. exp “Patient Acceptance of Health Care”/ 2. access*.mp. 3. adher*.mp. 4. attend*.mp. 5. complet*.mp. 6. complian*.mp. 7. drop?out.mp. 8. finish*.mp. 9. non?adher*.mp. 10. non?attend*.mp. 11. non?complian*.mp. 12. participat*.mp. 13. utilisation*.mp. 14. engagement*.mp. 15. uptake*.mp. 16. 45 or 46 or 47 or 48 or 49 or 50 or 51 or 52 or 53 or 54 or 55 or 56 or 57 or 58 or 59 | 1. (MH “Patient Compliance+”) 2. (MH “Health Services Accessibility”) 3. “access*” 4. “adher*” 5. “attend*” 6. “complet*” 7. “complian*” 8. (MH “Patient Dropouts”) 9. “drop#out” 10. “finish*” 11. “non#adher*” 12. “non#attend*” 13. “non#complian*” 14. “participat*” 15. (MH “Health Resource Utilization”) 16. “utili?ation*” 17. “engagement*” 18. “uptake*” 19. S46 OR S47 OR S48 OR S49 OR S50 OR S51 OR S52 OR S53 OR S54 OR S55 OR S56 OR S57 OR S58 OR S59 OR S60 OR S61 OR S62 OR S63 | 1. patient attitude/ 2. access*.mp. 3. adher*.mp. 4. attend*.mp. 5. complet*.mp. 6. complian*.mp. 7. drop?out.mp. 8. finish*.mp. 9. non?adher*.mp. 10. non?attend*.mp. 11. non?complian*.mp. 12. participat*.mp. 13. utilisation*.mp. 14. engagement*.mp. 15. uptake*.mp. 16. 42 or 43 or 44 or 45 or 46 or 47 or 48 or 49 or 50 or 51 or 52 or 53 or 54 or 55 or 56 | 1. exp “Patient Acceptance of Health Care”/ 2. access*.mp. 3. adher*.mp. 4. attend*.mp. 5. complet*.mp. 6. complian*.mp. 7. drop?out.mp. 8. finish*.mp. 9. non?adher*.mp. 10. non?attend*.mp. 11. non?complian*.mp. 12. participat*.mp. 13. utilisation*.mp. 14. engagement*.mp. 15. uptake*.mp. 16. 45 or 46 or 47 or 48 or 49 or 50 or 51 or 52 or 53 or 54 or 55 or 56 or 57 or 58 or 59 |
| 1. exp Socioeconomic Factors/ 2. deprivation*.mp. 3. disparit*.mp. 4. disadvantage*.mp. 5. education*.mp. 6. employ*.mp. 7. economic* marginali*.mp. 8. "food *secur*".mp. 9. housing*.mp. 10. "*income*".mp. 11. Neighborhood Characteristics/ 12. neighbo?rhood*.mp. 13. poverty.mp. 14. social class*.mp. 15. “*privilege*”.mp. 16. occupation*.mp. 17. socio?economic*.mp. 18. 61 or 62 or 63 or 64 or 65 or 66 or 67 or 68 or 69 or 70 or 71 or 72 or 73 or 74 or 75 or 76 or 77 | 1. (MH “Socioeconomic Factors”) 2. (MH “Sociodemographic Factors”) 3. (MH “Social Class”) 4. (MH “Healthcare Disparities”) 5. (MH “Health Status Disparities”) 6. “deprivation*” 7. “disparit*” 8. (MH “Social Deprivation”) 9. “disadvantage*” 10. “education*” 11. (MH “Employment Status”) 12. “employ*” 13. “economic* marginali*” 14. “food *secur*" 15. (MH “Food Security”) 16. “housing*” 17. “*income*” 18. (MH “Neighborhood Characteristics”) 19. “neighbo#rhood*” 20. “occupation*” 21. (MH “Poverty”) 22. “poverty” 23. “social class*” 24. “socio#economic*” 25. “*privilege*” 26. S65 OR S66 OR S67 OR S68 OR S69 OR S70 OR S71 OR S72 OR S73 OR S74 OR S75 OR S76 OR S77 OR S78 OR S79 OR S80 OR S81 OR S82 OR S83 OR S84 OR S85 OR S86 OR S87 OR S88 OR S89 | 1. socioeconomics/ 2. deprivation*.mp. 3. health disparity/ 4. health care disparity/ 5. disadvantage*.mp. 6. education*.mp. 7. employment/ 8. employ*.mp. 9. economic* marginali*.mp. 10. food security/ 11. "food *secur*".mp. 12. housing*.mp. 13. "*income*".mp. 14. neighborhood characteristic/ 15. neighbo?rhood*.mp. 16. occupation/ 17. occupation*.mp. 18. poverty.mp. 19. social class/ 20. socio?economic*.mp. 21. “*privilege*”.mp. 22. disparit*.mp. 23. social class*.mp. 24. 58 or 59 or 60 or 61 or 62 or 63 or 64 or 65 or 66 or 67 or 68 or 69 or 70 or 71 or 72 or 73 or 74 or 75 or 76 or 77 or 78 or 79 or 80 | 1. exp Socioeconomic Factors/ 2. deprivation*.mp. 3. disparit*.mp. 4. disadvantage*.mp. 5. education*.mp. 6. employ*.mp. 7. economic* marginali*.mp. 8. Social Class/ 9. housing*.mp. 10. "*income*".mp. 11. "food *secur*".mp. 12. Neighborhood Characteristics/ 13. neighbo?rhood*.mp. 14. occupation*.mp. 15. Poverty/ 16. poverty.mp. 17. social class*.mp. 18. socio?economic*.mp. 19. “*privilege*”.mp. 20. 61 or 62 or 63 or 64 or 65 or 66 or 67 or 68 or 69 or 70 or 71 or 72 or 73 or 74 or 75 or 76 or 77 or 78 or 79 |
| 1. 38 and 44 and 60 and 78 | 1. S38 AND S45 AND S64 AND S90 | 1. 35 and 41 and 57 and 81 | 1. 38 and 44 and 60 and 80 |
